# Tendon Anomaly Identification in Prestressed Concrete Beams Based on an Advanced Monitoring MEMS and Data-Driven Detection of Structural Damage

**DOI:** 10.3390/s25010289

**Published:** 2025-01-06

**Authors:** Giorgio de Alteriis, Giulio Mariniello, Tommaso Pastore, Alessia Teresa Silvestri, Giuseppe Augugliaro, Ida Papallo, Canio Mennuti, Antonio Bilotta, Rosario Schiano Lo Moriello, Domenico Asprone

**Affiliations:** 1 Department of Industrial Engineering, University of Naples Federico II, Piazzale Tecchio 80, 80125 Naples, Italy; rschiano@unina.it; 2 Department of Structures for Engineering and Architecture, University of Naples Federico II, Via Claudio 21, 80125 Naples, Italy; giulio.mariniello@unina.it (G.M.); tommaso.pastore@unina.it (T.P.); antonio.bilotta@unina.it (A.B.); d.asprone@unina.it (D.A.); 3 Department of Chemical, Materials and Production Engineering, University of Naples Federico II, Piazzale Tecchio 80, 80125 Naples, Italy; alessiateresa.silvestri@unina.it; 4 National Institute for Accident Insurance at Work (INAIL), Monte Porzio Catone, 00040 Rome, Italy; g.augugliaro@inail.it (G.A.); c.mennuti@inail.it (C.M.); 5 Department of Electrical Engineering and Information Technology, University of Naples Federico II, Via Claudio 21, 80125 Naples, Italy; ida.papallo@unina.it

**Keywords:** distributed monitoring system, structural health monitoring, MEMS, frequency domain decomposition, anomaly detection

## Abstract

The growing importance of state assessments in civil engineering has led to intensive research into the development of damage identification methods based on vibrations. Natural frequencies and modal shapes have garnered great interest because modal parameters are invariant of structure. Moreover, thanks to the global nature of modal parameters, their variations are not limited to the location of the damage. This is an important advantage that offers the opportunity to identify damage with sensors whose position does not have to coincide with the damaged area. The integration of MEMS sensors into structural health monitoring (SHM) systems offers a promising approach to long-term structural maintenance, especially in large-scale infrastructure. This paper presents an anomaly detection technique that analyzes raw sequential data within a statistical framework to detect damage that causes prestress loss of the tendon by exploiting a distributed monitoring system composed of six high-performance MEMS sensors. The proposed system is preliminarily evaluated to identify the frequency of the first mode, and then the proposed methodology is validated on acceleration data collected on a 240 cm beam in three different damage configurations, achieving a high detection accuracy and showing that its output can also evaluate the damage localization.

## 1. Introduction

The growing importance of structural condition assessments in civil engineering has prompted extensive research into the development of damage identification methods that utilize vibration measurements. Modal parameters, including natural frequencies, mode shapes, and modal damping, have emerged as critical indicators due to their invariance in spite of structural changes. These parameters reliably indicate the presence of damage, as structural changes manifest as variations in their values. Furthermore, their global nature enables the detection of damage without the need for sensors to be placed directly in the damaged area [1]. Recent advances in system identification, such as output-only stochastic subspace algorithms, have strengthened the focus on modal parameters further by providing numerically stable and reliable methods for experimentally determining these parameters from ambient excitations.

Structural health monitoring (SHM) is a crucial aspect of ensuring the safety and longevity of structures by providing key insights into their stress state and detecting the onset of damage. SHM encompasses a variety of techniques and technologies that have been widely adopted in both aerospace and civil engineering applications [2,3,4,5,6]. In infrastructure asset management, the safety of the structure is of critical importance, necessitating prompt and accurate maintenance interventions based on a comprehensive understanding of the structural behavior, health conditions, and traffic load data. This is of particular importance in the context of bridges, where it is essential to maintain low vulnerability levels in order to guarantee their safety and operational efficiency.

Damage and failure modes in complex systems like bridges can present in diverse ways. For prestressed concrete structures (PSCs), failure of the prestressing system is among the most critical types of damage, as it exhibits brittle behavior with little to no warning, potentially leading to catastrophic collapse [7].

Prestressed concrete has been widely adopted in engineering since it enables the realization of large-span elements. Specifically, this structural typology has largely been used in bridge construction. Nevertheless, this type of construction is particularly sensitive to degradation, and tendon rupture can result in fragile failure. Therefore, maintenance and surveillance of these structures are very compelling tasks, requiring continuous inspections and monitoring. The early detection of damage is essential to avoid sudden failures, ensuring user safety and reducing the financial burden of reactive maintenance or emergency repairs. In the detection of prestressing problems, it is a widely discussed topic in the literature [3,8,9], but it has not yet been solved with a unified solution. Therefore, it is necessary to evaluate techniques and methodologies that can detect damage, especially at an early stage.

In fact, by adopting monitoring systems, it will become possible to avoid inconvenience to users and, in the most extreme cases, to safeguard their safety. This can be achieved either through rapid intervention in cases of anomalies or adopting a Bridge Management System (BMS) for the optimization of maintenance activities that takes into account the structural condition [10]. Additionally, these systems can be integrated into overall frameworks with multiple goals, such as reducing one’s footprint [11] or ensuring structural robustness [12].

Therefore, the main outcome of this research is a method for the interpretation of monitoring data to be implemented in the workflows of entities managing reinforced concrete structures.

SHM systems offer reliable and real-time assessments of the condition of monitored structures [13,14,15]. SHM systems typically fall into two categories based on the data collected: static and vibration monitoring. Static monitoring, which tracks parameters such as displacements, deformations, and rotations, is widely used for monitoring damage evolution [16,17,18]. Although static methods for detecting tendon damage have been explored, they often require costly measurement technologies, such as fiber Bragg gratings [19], or emerging systems that are still under validation [9].

In recent years, Micro-Electro-Mechanical System (MEMS) sensors have increasingly been used in SHM systems. MEMS sensors, which are compact, low-cost, and low-power devices, offer the ability to measure a range of physical quantities, such as acceleration, strain, pressure, and temperature, with high accuracy [14,17,20,21,22]. Their integration into SHM systems provides several advantages, including improved data acquisition and processing capabilities, enhanced reliability, and reduced power consumption. These sensors enable real-time monitoring of stress states, early damage detection, and the prediction of structural lifespans. In bridge applications, MEMS sensors have demonstrated the ability to continuously monitor changes in structural conditions caused by external loads, traffic, or environmental factors. Despite these benefits, their limitations, such as noise and sensitivity to environmental changes, necessitate careful consideration. For instance, random noise and bias drift can complicate error compensation and limit the long-term applicability of inertial MEMS sensors [23,24]. Addressing these challenges requires attention to the sensor classification based on bias instability and random walk parameters [25].

Traditional SHM methods, such as strain gauges and wired sensor networks, have long been valued for their reliability and precision. However, these methods often encounter challenges, including high installation costs, limited scalability, and vulnerability to environmental interference. MEMS technology offers a promising alternative, with advantages such as compactness, high sensitivity, and wireless operation capabilities. While traditional accelerometers excel in high-frequency vibration monitoring, MEMS sensors perform well in low-frequency applications and integrate seamlessly into wireless sensor networks. The recent literature has highlighted innovations in MEMS technology, including advancements in smart sensors for SHM [26], critical reviews of embedded sensors, and improvements in sensor durability in high-temperature environments [27].

MEMS sensors integrate mechanical and electrical components at the microscale to measure physical parameters such as acceleration and displacement. Their working principles, based on changes in capacitance, translate structural responses into electrical signals for analysis [28,29]. Their small size and low power requirements make them ideal for deployment in challenging environments, including prestressed concrete structures. Furthermore, their wireless capabilities eliminate extensive cabling and facilitate real-time data acquisition over large areas. These features make MEMS sensors particularly effective in detecting early signs of structural deterioration, such as crack propagation and shifts in modal frequencies. However, challenges such as their temperature sensitivity and long-term degradation remain significant, driving ongoing research into enhancing sensors’ durability and developing algorithms to mitigate environmental effects [30].

By highlighting the capabilities and limitations of MEMS sensors in SHM, particularly for prestressed concrete structures, this discussion underscores their potential to transform modern infrastructure monitoring. The integration of MEMS-based solutions represents a significant advancement in SHM methodologies, providing comprehensive, scalable, and cost-effective tools for ensuring the safety and longevity of critical infrastructure.

Notwithstanding these challenges, MEMS sensors have the potential to advance SHM systems significantly. They offer engineers and end users efficient, accurate, and cost-effective solutions for structural monitoring and maintenance. Continued advancements in MEMS technology and data analytics are expected to enhance the capabilities of SHM systems further, thereby inaugurating a novel approach to structural monitoring and maintenance practices [2].

Vibration monitoring captures the dynamic behavior of a structure under operational and ambient conditions using velocimeters or accelerometers. The data obtained can be analyzed directly or employed in an Operational Modal Analysis (OMA) to determine the dynamic properties of a structure [31]. This approach provides a global assessment of the structure; however, it may be less effective in detecting early-stage or localized damages, such as tendon failures, which may not significantly affect its dynamic properties [32].

The primary aim of this research is to develop and validate an anomaly detection method using MEMS-based sensors to monitor the tendon integrity in prestressed concrete structures. This study is grounded in vibration-based methods, which allow for the detection of structural changes not directly adjacent to the sensor placement.

## 2. The Proposed Method

The early detection of damage in prestressing systems is critical for ensuring the safety and reliability of PSC structures. The methodology proposed in this paper leverages a similarity analysis of acceleration time histories (i.e., sequences of acquired acceleration samples) to distinguish between healthy and damaged structural states. The approach is divided into two phases: the calibration phase, in which reference data from the undamaged structure are collected, and the operational phase, in which new data are analyzed to assess structural health.

During the calibration phase, multiple acceleration time histories are collected to represent the structure’s dynamic behavior in its undamaged state. These data will be employed to define the baseline conditions and represent the reference for anomaly detection. The duration of these histories depends on the SHM system’s capabilities, with 600 s histories used in this study. Let *K* be the number of accelerometers installed on the structure to be monitored and *N* the number of histories acquired using each accelerometer during the calibration stage. The data from the accelerometers are arranged into a reference matrix of acceleration time histories ($Acc^{ref}$), where $Acc_{i_{0},j_{0}}$ represents the $i_{0}$-th time history of the $j_{0}$-th accelerometer.

(1) $$Acc^{ref}=\left(\begin{array}{ccc} Acc_{1,1} & \cdots & Acc_{1,N}\\ \vdots & \ddots & \vdots \\ Acc_{K,1} & \cdots & Acc_{K,N} \end{array}\right)$$

The similarity between time histories is measured by means of a proper function, i.e., the Minimum Jump Cost (MJC), as discussed by Serra and Arcos [33]. By calculating the mean similarity index between each $Acc_{i_{0},j_{0}}$ and all the other time histories from the same sensor, a similarity index (SI) matrix is constructed. The generic element of the SI matrix is defined as follows:

(2) $$SI_{i_{0},j_{0}}=\frac{\sum_{i\;\neq i_{0}}Sim(Acc_{i_{0},j_{0}}^{ref};Acc_{i,j_{0}}^{ref})}{N-1}$$

To detect anomalies, the methodology applies a control chart approach, one of the most widespread anomaly detection techniques. The mean (μ) and standard deviation (σ) of the SI matrix are computed, and the threshold values are defined as follows:

(3) μ±λ σ

These threshold values are derived from Chebyshev’s theorem [34], which provides an upper bound on the probability of observing values outside a specified range. When λ = 3, and assuming no significant changes in the bridge’s structural behavior, Chebyshev’s theorem dictates that the probability of the measurements falling outside the defined thresholds is at most 11%. The calculation of these thresholds completes the preliminary calibration phase. During the operational phase, the system periodically collects acceleration data to monitor the bridge’s condition. For each new set of measurements, the similarity index is calculated by comparing the time histories of the new data to the reference dataset ($Acc^{ref}$). The generic element of the new similarity index matrix is expressed as follows:

(4) $$SI_{{i0,j}_{0}}^{new}=\frac{\sum Sim(Acc_{i_{0},j_{0}}^{new};Acc_{i,j_{0}}^{ref})}{N}$$

For each time history ($j_{0}$), if even a single element of the new similarity index matrix $SI_{j_{0}}^{new}$ falls outside the defined thresholds, the system identifies it as an outlier. According to Chebyshev’s theorem, when the proportion of outliers exceeds 11%, the system detects an anomaly in the structural behavior and triggers an alert.

**Figure 1:**
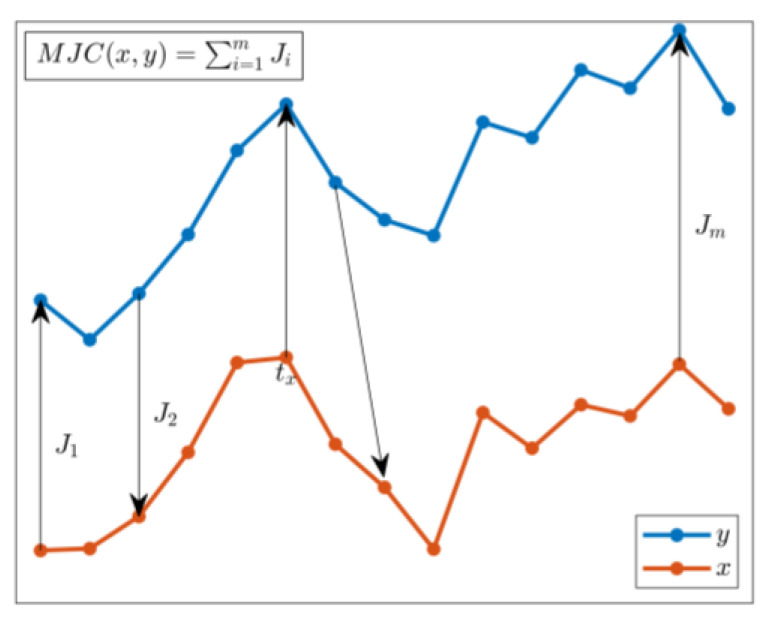
Minimum Jump Cost description.

The similarity index adopted to compare time history is the MJC function, which is evaluated by calculating the cost obtained by jumping from one time history to another. In particular, given two arbitrary sequences, x and y, the MJC is calculated by sequentially traversing their data points while enforcing unidirectional forward progression. Starting from an arbitrary abscissa $t_{x}$ in sequence x, the MJC evaluates all possible jumps to the last component of sequence y, selecting the most optimal jump to reach point $t_{y}$. The process then continues by identifying the next jump from $t_{y+1}$ in sequence y back to sequence x. This iterative procedure is repeated until one of the sequences concludes, with the resulting MJC being the sum of all selected jumps (Figure 1) and, according to Equation (5), $J_{i}$ being the cost difference between the two time histories (x and y). For further methodological details and the formalism underpinning the MJC, please refer to [33].

(5) $$MJC\left(x,y\right)=\sum_{i=1}^{m}J_{i}$$

In this work, the Minimum Jump Cost (MJC) measure is adopted as the criterion for comparing two distinct time histories, i.e., for evaluating the distance between them. However, alternative approaches can be employed for defining the distance measure. In fact, Equation (2) introduces a generic notation, *Sim*, to represent any arbitrary similarity measure, emphasizing the flexibility of the proposed method.

### 2.1. System Architecture

**Figure 2:**
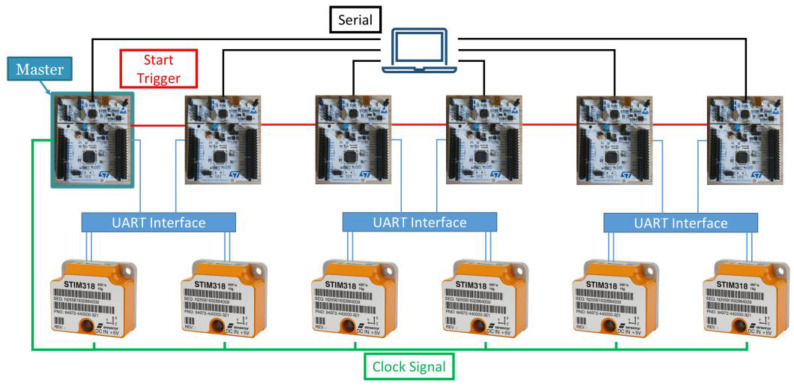
System architecture based on six high-grade MEMS sensors.

The data acquisition system, which utilizes MEMS sensors, is illustrated in Figure 2. This system comprises six high-performance inertial sensors, manufactured by Sensonor™ (Skoppum, Norway), and six microcontrollers, manufactured by STMicroelectronics™ (Geneva, Switzerland). In this configuration, the microcontrollers, specifically the STM32F446RE, collect data from the six STIM318 [35] sensors via the UART protocol. To facilitate this communication, an interface between the RS422 and UART protocols was implemented using the SN75C1167 chip from Texas Instruments (Dallas, TX, USA); in this way, the microcontrollers acquire data from the UART.

For system synchronization, data from the STIM318 sensors are sampled using an external clock provided by the Master microcontroller, i.e., one of the six microcontrollers. The STIM318 sensors are configured to sample data at 2 kHz, while the triggering frequency is set to 250 Hz, thereby limiting the average delay between the data request and actual sampling to 250 μs. Moreover, the Master microcontroller generates a start signal, which is captured by the other microcontrollers to ensure the synchronized start of the data acquisition process. Finally, the data are sent to a PC, which are used to trigger acquisition by the Master and collect data from all of the microcontrollers by means of serial communication.

The architecture for this system was chosen based on several critical considerations, which collectively contribute to its effectiveness in structural health monitoring applications. First, the system incorporates high-performance sensors and microcontrollers. The STIM318 sensor supports high-frequency data acquisition at 2 kHz, with a noise density equal to 0.015 $m/s/\sqrt{hr}$. The microcontroller, an Arm Cortex-M4 running at 180 MHz, complements the sensor’s capabilities by ensuring efficient processing of the collected data. Second, synchronization capabilities were a fundamental factor in the selection process. Accurate synchronization between the sensors and the microcontroller is essential for structural monitoring to ensure consistent and reliable data collection across multiple sensors. This is achieved through the external clock and the start signal provided by the Master’s microcontroller, which minimizes delays and enhances the reliability of the acquired data. Another key consideration was communication reliability. The system employs an RS422-to-UART interface, which ensures robust and efficient data transmission between the sensor and the microcontroller. Lastly, the modularity and scalability of the architecture played an important role in its selection. The system is designed to adapt to various structural elements or accommodate additional sensors as needed. Furthermore, its design allows for integration with Internet of Things (IoT) protocols, making it a flexible and forward-compatible solution suitable for a wide range of SHM applications.

### 2.2. Measurement Setup

**Figure 3:**
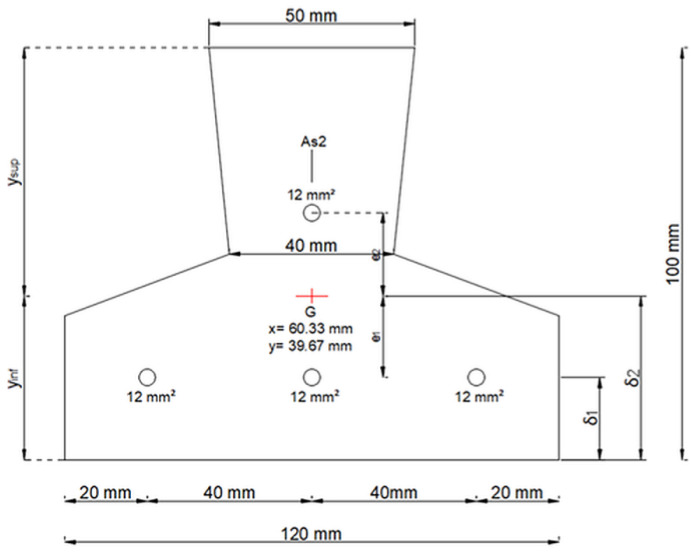
Depiction of the joist section and tendon layout.

The proposed methodology was tested through an experimental case study involving a prestressed concrete joist, a structural element commonly employed in floor construction. The joist used in the study measured 240 cm in length and had a T-shaped cross-section, with a height of 10 cm, a major base width of 12 cm, and a minor base width of 5 cm. The reinforcement configuration consisted of three prestressing tendons located in the lower section and one tendon in the upper section, each with a cross-sectional area of 12 mm^2^. The joist was constructed using C45/55 class concrete, characterized by a cylindrical compressive strength (f_ck_) of 45.65 N/mm^2^ and an elastic modulus (EEE) of 36,416.11 N/mm^2^. The tendons were made of harmonic steel, which possesses a characteristic yield stress (f_p(1)k_) of 1670 N/mm^2^ and a characteristic ultimate tensile strength (f_ptk_) of 1860 N/mm^2^. A detailed representation of the cross-section of the joist is provided in Figure 3, while the characteristics of the concrete and the tendons are reported in Table 1.

**Figure 4:**
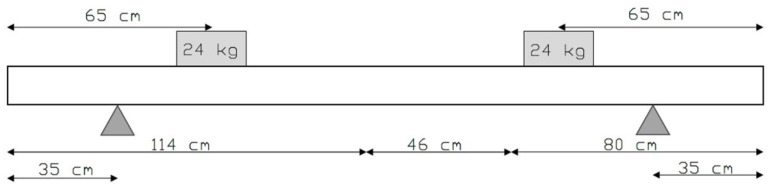
Setup for the experiments.

The experimental setup, as illustrated in Figure 4, involved placing the joist specimen onto two symmetrical vertical supports, positioned 35 cm from each end of the beam. To simulate loading conditions, two masses of 24 kg each were placed 65 cm from the respective ends of the beam, ensuring an evenly distributed load across the structure.

The proposed monitoring system was also preliminarily evaluated by means of a comparison of measurements made using a low-noise MEMS sensor, i.e., an EPSON M-A352 accelerometer, which was considered a reference system [36]. The data acquisition was realized with an additional microcontroller that generated not only the clock signal but also operated as an interface between the sensor and the PC. The synchronization between the proposed monitoring system and the reference was realized with a digital start signal received from the Master microcontroller.

**Figure 5:**
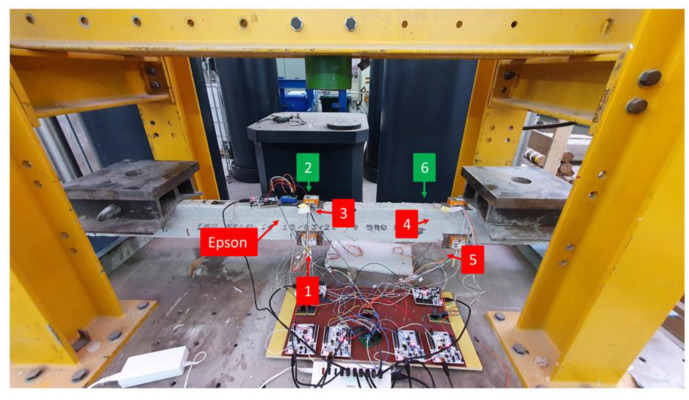
Distributed monitoring system of six STIM318 sensors (highlighted in red and green colors for front and back, respectively) in parallel configuration.

**Figure 6:**
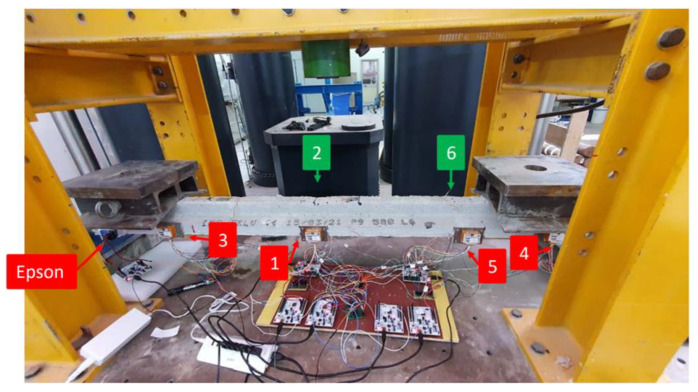
Distributed monitoring system of six STIM318 sensors (highlighted in red and green colors for front and back, respectively) in longitudinal configuration.

The aforementioned sensors were arranged considering two types of configurations:

1. A parallel configuration, where the STIM sensors were positioned in sets of 3 in two sections located 114 cm and 160 cm from the left end of the beam. In each section, one STIM sensor was placed on the front side, one on the rear side, and one on the top side. The EPSON sensor instead was positioned on the top side of the beam 110 cm from the left end (near STIM sensor 3). Figure 5 illustrates the arrangement of the sensors:
2. A longitudinal configuration, where four STIM sensors and the EPSON sensor were placed on the front side of the beam, and two STIM sensors were placed on the rear side (Figure 6).

Optimization of the accelerometers’ layout is a relevant topic in the field of SHM; several authors have suggested methods and strategies for optimizing the positioning to improve the detection in order to improve the identification of the dynamic properties of structures [37]. Although defining the optimal location is outside the scope of this paper, the authors adopted two different configurations to evaluate their performance in dynamic identification. Conversely, the damage detection analyses, on the other hand, were conducted using only the parallel configuration, which provided greater sensitivity to off-center damage, which may induce a change in internal stress and thus beam deflection, such as in a clipped tendon.

## 3. System Characterization

### 3.1. Measurement Synchronization

**Figure 7:**
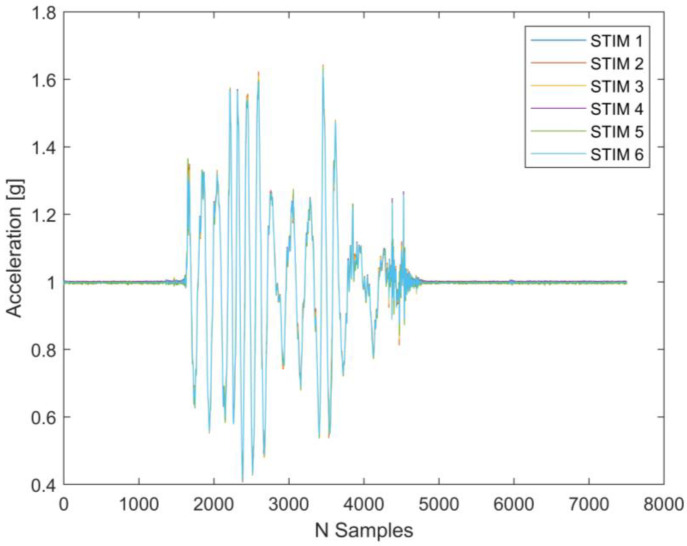
Comparison of acceleration measurements of all sensors under controlled displacements in one direction.

**Figure 8:**
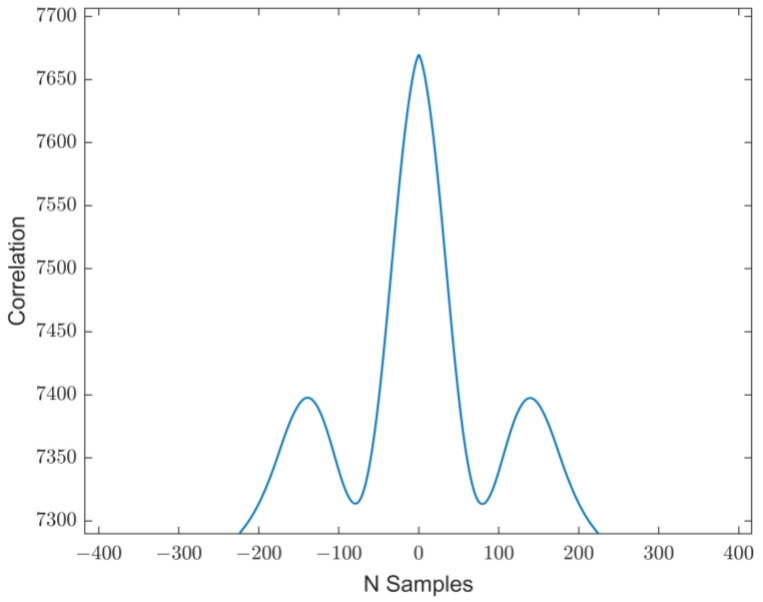
Correlation of two acceleration measurements.

The distributed monitoring system was initially tested through controlled displacements in a single direction to evaluate the proper synchronization of the acquired acceleration signals (Figure 7). In this context, the correlation between the signals was analyzed, and the result of combining two signals is presented in Figure 8.

The cross-correlation between two acceleration signals is computed using MATLAB’s *xcorr* function (version: 2024b). The *x*-axis represents the lag in terms of the sample shifts (N samples), while the *y*-axis corresponds to the correlation values. The peak of the correlation occurs at lag = 0, which suggests a high level of synchronization between the two signals without any relative time delay. Additionally, the symmetrical nature of the curve indicates that the signals are temporally aligned, with no significant phase shift. The side lobes on either side of the main peak reflect secondary correlations due to periodic or repeating patterns in the signals. This result validates the synchronization of the two signals, which arise from shared, similar dynamic responses.

Additionally, the time required to retrieve data from the sensor, as well as the transmission and reception times for the acquisition start signal, were measured. The mean value for data retrieval was found to be 0.54 ms, with a standard deviation of approximately 500 ns, while the transmission and reception times were around 40 ns, respectively. Furthermore, the microcontroller’s internal timers were utilized to measure the actual sampling rate, where a value of 4 ms was obtained.

### 3.2. Performance Evaluation

Frequency Domain Decomposition (FDD) is an output-only method that proves valuable in discerning the vibration frequencies and corresponding modal shapes of a structural system from the accelerations recorded in the structure. The essence of this technique lies in the premise that the eigenvectors, representing the vibration modes, constitute a basis due to their linear independence. Consequently, any system displacement can be expressed as a linear combination of these eigenvectors, facilitating decoupling of the mode components. This property finds application in analyzing the system’s response, observed at each accelerometer placement point, or in examining the Power Spectral Density (PSD) of the accelerometer’s history through Singular Value Decomposition (SVD) of the matrix defined for each frequency ω [38].

This method leverages frequency response functions (FRFs) to extract the eigenperiods, damping, and modal deformations of the structure. Specifically, Fourier transform aids in converting the dynamic behavior governing the differential equations of the structure into a system of algebraic equations for easier resolution.

**Figure 9:**
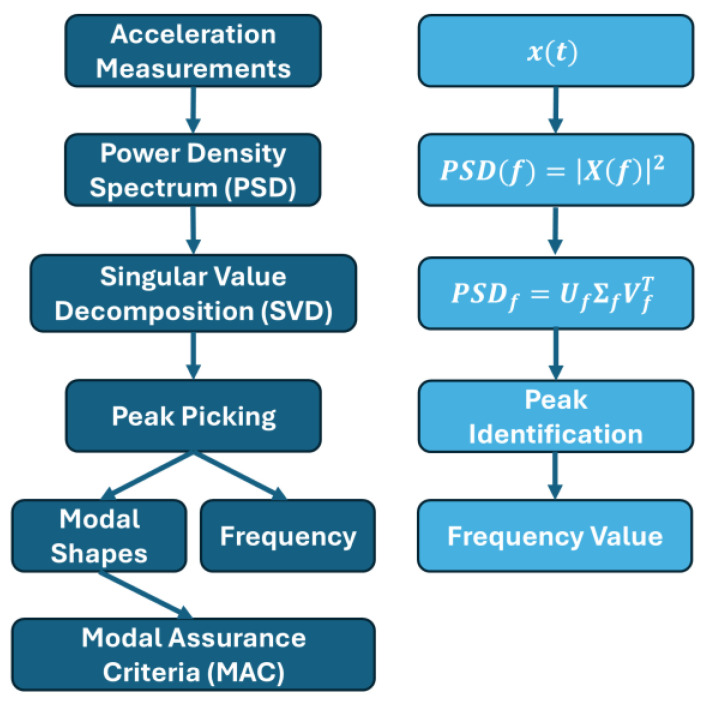
FDD technique flowchart.

Figure 9 depicts a flowchart illustrating the step-by-step data processing procedure via the FDD technique as a description and a mathematics equation. Initially, the PSDs are estimated through Fourier transforms of the signals $x\left(t\right),$ which are the means of the acceleration measurements. Subsequently, the PSD matrices—with one for each frequency f—undergo decomposition into singular values, where $U_{f}$ and $V_{f}$ are the singular vectors, while $\Sigma_{f}$ are the singular values. These singular values depict the degrees of the structural system, while the singular vectors carry information on the modal form. Vibration modes are then identified based on the graphical representation of the singular value spectrum at resonance peaks. Natural frequencies are visually discerned using the peak-picking technique. Each peak identified corresponds to a singular value, matched with a singular vector. The appropriateness of attributing a frequency to a vibration mode is assessed through the Modal Assurance Criteria (MAC) [39,40]. It is therefore essential to evaluate the frequency in order to identify the component of interest. To this end, the proposed system was characterized through comparison with the results obtained using the Artemis Modal Pro software (version: 8) and the reference system.

In particular, Table 2 shows the frequencies obtained by means of the software, which identifies that the first mode exhibits frequencies of about 50 Hz and 53 Hz in the absence of an applied load for the parallel and longitudinal configurations, respectively, while frequencies of about 40 Hz and 41 Hz for the parallel and longitudinal configurations, respectively, are observed with an applied load, a result that is also evident in the finite element model. For the unloaded beam, the discrepancies between the FEM and Artemis, ranging between 5% and 10%, likely stem from minor differences in the boundary conditions and material properties inherent to the theoretical and experimental setups.

**Figure 10:**
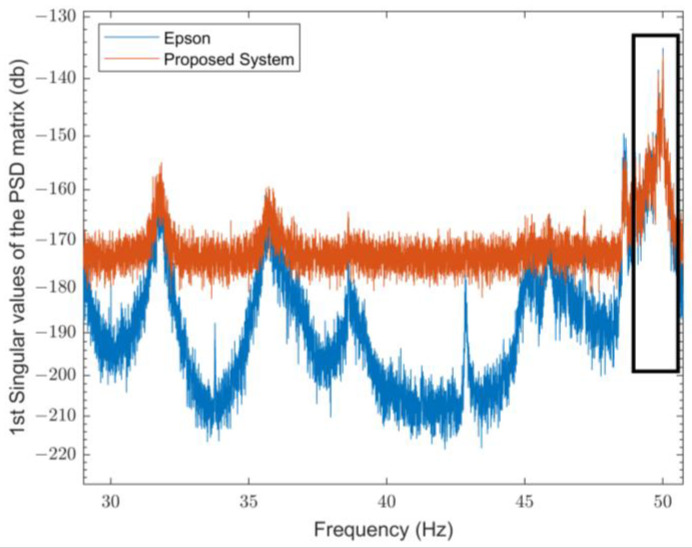
First mode of the parallel configuration without applied load.

**Figure 11:**
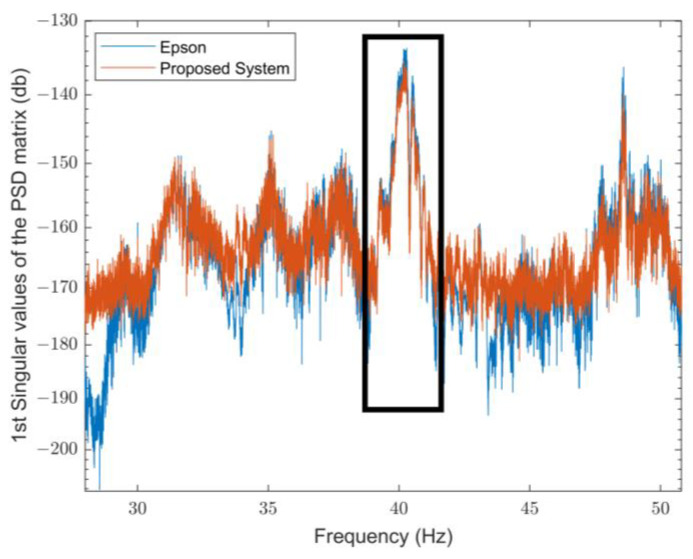
First mode of the parallel configuration with applied load.

On the other hand, in the presence of a load on the analyzed beam, the differences are slightly more pronounced, at around 20%. This variation can be attributed to the influence of the applied load, which affects the stiffness and stress distribution. These factors are more accurately reflected in the experimental conditions than in the FEM’s idealized assumptions. Once the first mode had been evaluated through the FEM analysis and using Artemis Modal Pro software, the outcomes obtained by the proposed system and the reference system were then examined around the frequency of interest, as shown in Figure 10 and Figure 11 for the parallel configuration in the absence of an applied load and in the presence of an applied load, respectively. Both systems correctly identify the frequency of the first mode, with values equal to 50.01 Hz for the reference system and equal to 50.01 Hz for the proposed system for an unloaded beam and values equal to 40.29 Hz for the reference system and equal to 40.18 Hz for the proposed system in the loaded case. Furthermore, both systems demonstrate a variation in the frequency of the first mode of the beam from about 50 Hz to 40 Hz when a load is applied, i.e., in dynamic conditions.

## 4. Results

**Figure 12:**
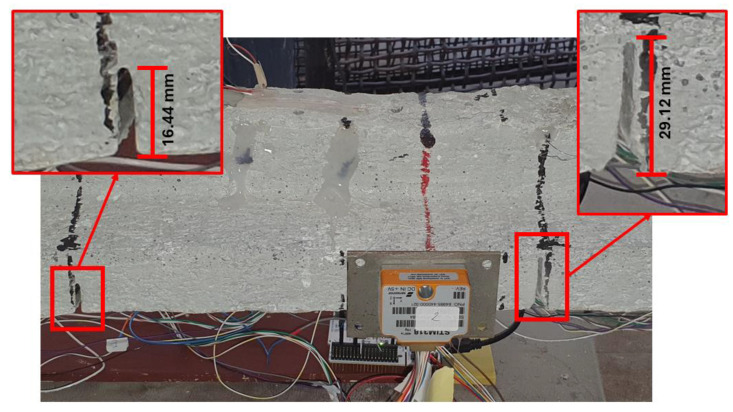
Example of cuts near the tendons.

**Figure 13:**
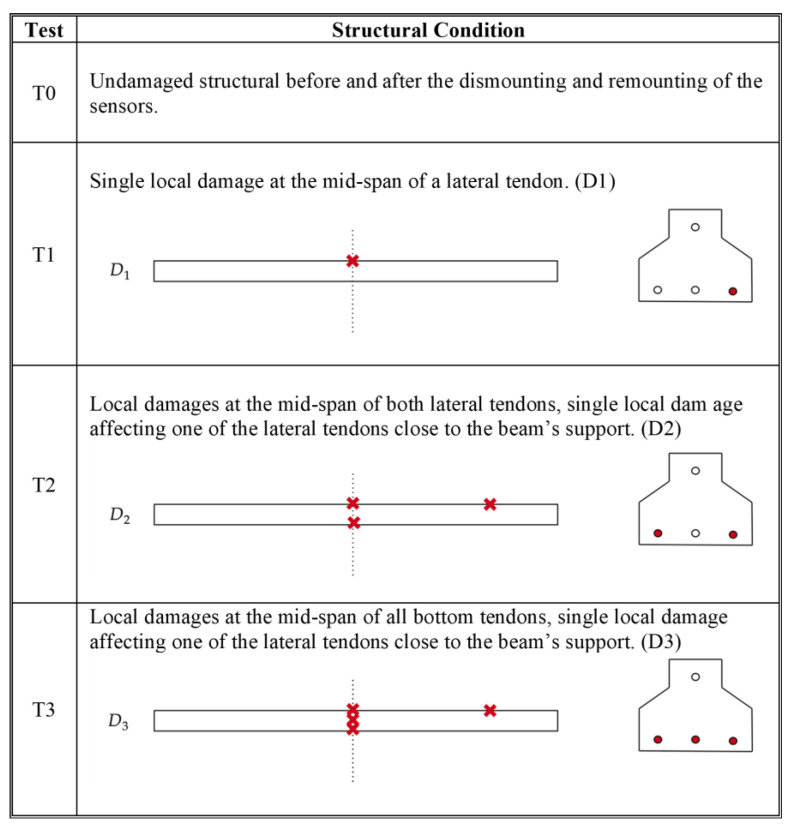
Experimental damage patterns adopted.

The experimental campaign consisted of four tests (T0–T3), with each aiming to evaluate both the identification system’s performance under an increasing damage severity and the effectiveness of the proposed methodology for identifying tendon failure. All of the experimental tests involved the acquisition of 146 time histories of 600 s in ambient conditions. The damage was induced by creating narrow, deep cuts near the tendons, as shown in Figure 12. In each test, the system’s abilities to detect anomalies and correlate the number of alerts with the damage extent were assessed. Test T0 served to confirm that the system does not trigger alerts in response to minor variations in the positions of the masses or sensors, while tests T1 to T3 assessed the system performance under the conditions of progressively increasing damage. This study further sought to explore the potential correlation between the number of alerts generated and both the extent and location of the damage. Figure 13 provides a detailed overview of the damage patterns applied in each experiment. For this campaign, the similarity function introduced previously, in the context of time-series analysis, was utilized.

The initial test evaluates the sensitivity of the methodology to minor changes in the experimental setup. Specifically, the response of the undamaged joist is assessed by disassembling and reassembling the sensors to determine the system’s sensitivity to slight modifications in the sensor–structure configuration. According to Chebyshev’s theorem, the control chart of the probability of having values outside the set thresholds indicates this is 11% at most, i.e., the anomalies can be detected according to the following:

(6) $$\frac{Number\;of\;outliers}{Number\;of\;samples}>0.11$$

The results of analyzing the outliers are shown in the following control charts, where they illustrate the performance across three data groups: training, testing, and validation. The training data depicted in blue are used to define the control chart thresholds. The validation set, indicated in green, is obtained from the data on the undamaged structure that were collected during the training phase. The testing group, represented in red, indicates the testing data.

**Figure 14:**
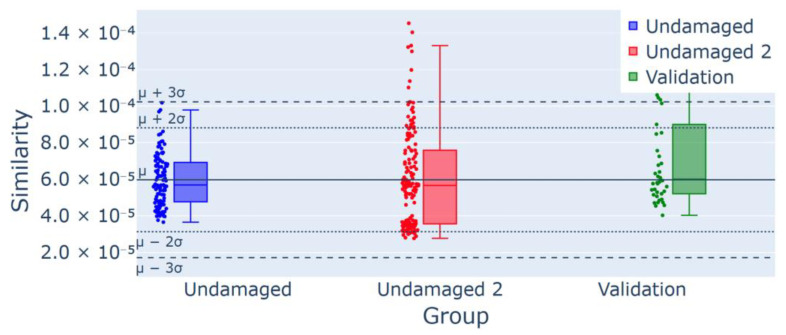
Control chart for experiment T0 for sensor arrangement: 8/146 (5.5%) outliers for the group Undamaged 2, confirmed using the validation group as a part of the undamaged data.

Figure 14 presents the results of the T0 damage detection analysis, showing the control chart derived from the similarity index under undamaged conditions. The chart includes data for the calibration-free validation measurements (green) and those made post sensor disassembly and reassembly (red). According to Figure 14, the number of outliers detected in the “Undamaged 2” dataset is 8, yielding an occurrence frequency of 5.5% for 146 five-minute time histories (approximately 12 h). This value remains below the 11% threshold required to trigger an alert, indicating the robustness of the system to minor sensor adjustments.

**Figure 15:**
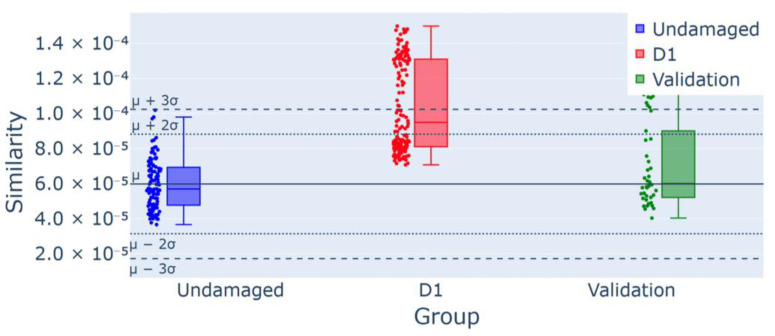
Control chart for experiment T1 for D1 damage detection: 64/146 (43.8%) outliers for the group D1, confirmed using the validation group as a part of the undamaged data.

The results from test T1, with the D1 damage scenario, are shown in Figure 15, where the similarity index computed for the time histories under D1 conditions is represented in red. The methodology detected 64 outliers, approximately 43.8% of the dataset, which exceeded the 11% threshold, leading to anomaly detection and indicating structural changes.

**Figure 16:**
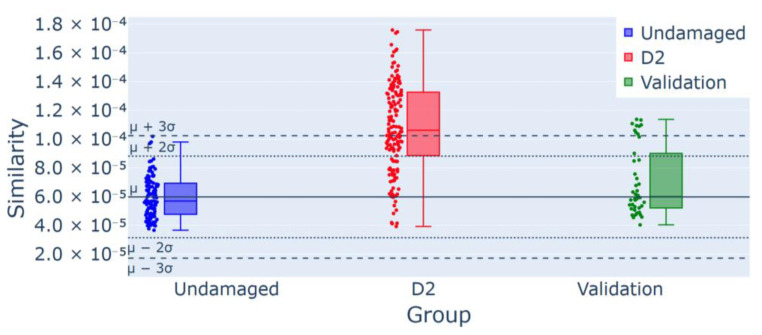
Control chart for experiment T2 for D2 damage detection: 80/146 (54.8%) outliers for the group D2, confirmed using the validation group as a part of the undamaged data.

**Figure 17:**
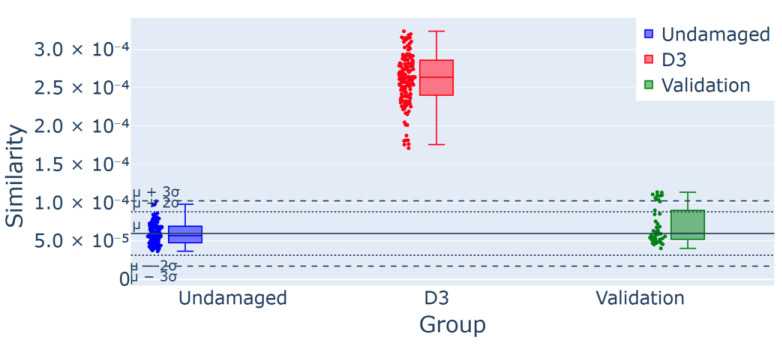
Control chart for experiment T3 for D3 damage detection: 146/146 (100%) outliers for the group D3, confirmed using the validation group as a part of the undamaged data.

In advanced damage scenarios (T2 and T3), as shown in Figure 16 and Figure 17, the control charts visually illustrate an increased number of outliers for damage scenarios D2 and D3, with the alerts totaling 80 and 146, respectively, corresponding to 54.8% and 100% of the newly collected measurements. These findings confirm the detection of anomalies in each case.

Regarding T2 and T3, the control charts in Figure 15 and Figure 17 already visually highlight a considerable number of outliers that result in the detection of an anomaly. In cases D2 and D3, there are 80 and 146 alerts, respectively, which consist of 54.8% and 100% of the sample of new measurements.

The system successfully detected damage in all scenarios (D1–D3) and demonstrated a direct correlation between the damage severity and the percentage of outliers. As the damage intensified, the percentage of outliers rose, reaching 100% in the most severe cases. Therefore, referring only to the investigated scenarios and adopting the 11% outlier threshold gives an overall accuracy of 100% because the presence or absence of damage was correctly detected in all four investigated scenarios.

**Figure 18:**
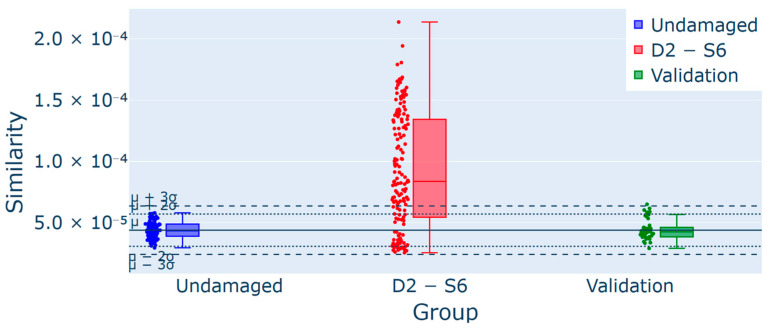
Control chart for experiment T2 for D2 damage detection from sensor 6 (S6): 99/146 (67.8%) outliers for the group D2-S6, confirmed using the validation group as a part of the undamaged data.

**Figure 19:**
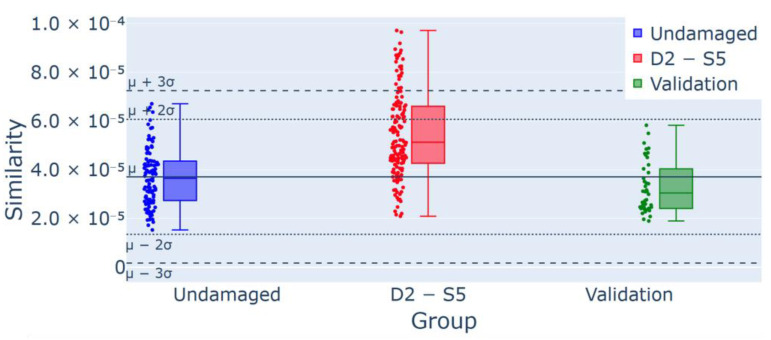
Control chart for experiment T2 for D2 damage detection from Sensor 5 (S5): 25/146 (17.1%) outliers for the group D2-S5, confirmed using the validation group as a part of the undamaged data.

A further analysis involved the detection of damage localization using a sensor-based analysis; in particular, this was conducted on test T2 by evaluating each sensor individually. Figure 18 shows that sensor 6, located near the damage, registered a higher number of outliers compared to sensor 5 (Figure 19), positioned on the opposite face of the same section. This suggests that the methodology may effectively localize damage within the structure. All of the experiments and the results they achieved are reported in Table 3, where the damage scenarios, key objectives, number of alerts, outlier percentages, and observations have been summarized.

## 5. Conclusions

In this research, the authors implemented a distributed monitoring system based on MEMS sensors. A preliminary evaluation of the system’s performance was conducted using a comparison with a low-noise sensor in a case study. This involved the use of initially unloaded and loaded prestressed concrete beams, with the objective of validating the proposed method. Subsequently, the results were also compared with those of analytical and finite element models. Finally, the authors proposed a damage detection framework for PSC beams based on a direct analysis of the acceleration time histories acquired from a reliable and affordable SHM system. Damage detection in prestressed bridge systems remains a key challenge in structural engineering. Reliable assessments are crucial to ensure transport safety. The authors proposed a methodology for identifying damage in prestressed concrete elements by analyzing the acceleration time histories using a similarity index to flag anomalies. Thresholds are established from a reference dataset, and measurements surpassing these limits are classified as outliers. Based on Chebyshev’s theorem, a structural anomaly is identified if the outliers exceed 11% of the measurements; lower values are considered within distributional variance.

The method was validated experimentally on a reinforced concrete joist across four tests. The initial test assessed robustness against false positives, revealing no anomalies when the sensor positioning was slightly altered. The subsequent tests targeted damage detection, with the outlier percentages ranging from 43% to 100%, indicating a strong detection accuracy and correlation between the frequency of outliers and the damage intensity. In the final test, the individual sensor analysis suggested the potential for damage localization using single-sensor data, where the sensors closest to the damage have a high number of outliers.

In conclusion, this methodology demonstrates robust performance across various damage levels in controlled conditions. Its primary limitations are the need for a com-prehensive baseline dataset and an adequate sensor count for multiscale damage detection. Future work will explore different similarity metrics and machine learning approaches to account for environmental variability.

## Figures and Tables

**Table 1:** Characteristics of concrete and tendons of harmonic steel.

Feature	Dimension	Value
$f_{ck}$	MPa	45.65
$E_{c}$	MPa	36,416.11
$E_{s}$	MPa	201,000
$f_{ptk}$	MPa	1860
$f_{p(1)k}$	MPa	1670

**Table 2:** First mode frequencies obtained with the FEM and Artemis Modal Pro.

	FEM	Artemis	
	[Hz]	Parallel Config. [Hz]	Longitudinal Config. [Hz]
Unloaded	55.16	50.68	53.83
Loaded	34.10	40.87	41.62

**Table 3:** Summary table of experiments and results obtained.

Test	Damage Scenario	Key Objective	Number of Alerts	Outlier Percentage	Observations
T0	None (Undamaged)	Validate system’s robustness to minor changes	8	5.50%	No alerts triggered; system insensitive to minor adjustments (sensor reassembly).
T1	D1 (Minor Damage)	Detect anomalies under minor damage	64	43.80%	System successfully detected damage; outlier percentage exceeded the 11% threshold.
T2	D2 (Moderate Damage)	Detect anomalies under moderate damage	80	54.80%	Increased outlier percentage confirms detection; correlation with damage severity observed.
T3	D3 (Severe Damage)	Detect anomalies under severe damage	146	100.00%	All samples flagged as outliers; clear indication of severe structural changes.
T2 (Only Sensor 5)	D2	Assess damage localization ability	25	17.10%	Sensor 6 near damage showed more alerts than sensor 5, suggesting effective damage localization.
T2 (Only Sensor 6)	D2	Assess damage localization ability	99	67.80%	

## Data Availability

Data available on request due to privacy restrictions.
